# Expansion of a novel population of NK cells with low ribosome expression in juvenile dermatomyositis

**DOI:** 10.3389/fimmu.2022.1007022

**Published:** 2022-10-31

**Authors:** Kinsey A. Hilliard, Allison A. Throm, Jeanette T. Pingel, Nermina Saucier, Hani S. Zaher, Anthony R. French

**Affiliations:** ^1^ Division of Pediatric Rheumatology/Immunology, Department of Pediatrics, Washington University School of Medicine, St. Louis, MO, United States; ^2^ Department of Biomedical Engineering, Washington University, St. Louis, MO, United States; ^3^ Department of Biology, Washington University, St. Louis, MO, United States

**Keywords:** NK cell, JDM, RNAseq, ribosome, autoimmunity, rheumatology, PBMCs, immune dysregulaiton

## Abstract

Juvenile dermatomyositis (JDM) is a pediatric autoimmune disease associated with characteristic rash and proximal muscle weakness. To gain insight into differential lymphocyte gene expression in JDM, peripheral blood mononuclear cells from 4 new-onset JDM patients and 4 healthy controls were sorted into highly enriched lymphocyte populations for RNAseq analysis. NK cells from JDM patients had substantially greater differentially expressed genes (273) than T (57) and B (33) cells. Upregulated genes were associated with the innate immune response and cell cycle, while downregulated genes were associated with decreased ribosomal RNA. Suppressed ribosomal RNA in JDM NK cells was validated by measuring transcription and phosphorylation levels. We confirmed a population of low ribosome expressing NK cells in healthy adults and children. This population of low ribosome NK cells was substantially expanded in 6 treatment-naïve JDM patients and was associated with decreased NK cell degranulation. The enrichment of this NK low ribosome population was completely abrogated in JDM patients with quiescent disease. Together, these data suggest NK cells are highly activated in new-onset JDM patients with an increased population of low ribosome expressing NK cells, which correlates with decreased NK cell function and resolved with control of active disease.

## Introduction

Juvenile dermatomyositis (JDM) is an inflammatory myopathy affecting striated muscle, skin, and the gastrointestinal tract with an incidence of 3.2 cases per million children (1). Patients experience proximal muscle weakness and exhibit characteristic skin findings including heliotrope rash, Gottron papules, calcinosis cutis, and periungual erythema & telangiectasias (2). Before the use of steroid therapy, JDM had a mortality rate of 40%. With current therapies, JDM has a mortality rate of less than 2% (3), but it still imposes significant morbidity with over 25% of JDM patients having persistent symptoms for over 3 years (4).

The etiology of JDM is not well understood, but it has been hypothesized that it involves a combination of environmental triggers, immune dysfunction, and specific tissue responses (2) with contributions from both defective adaptive and innate immune responses. Approximately 65% of JDM patients have myositis-specific or myositis-associated antibodies (3, 5), and B cell depletion with rituximab improves symptoms in some patients (6, 7) implicating a potential role for B cells. Furthermore, recent work identified an expansion of immature transitional B cells in JDM PBMCs (8). JDM patients have increased T cell skewing towards Th2 and Th17 responses, which correlates with disease activity (9). Dysregulation of T regulatory cells with restricted diversity has also been implicated in JDM (10). Innate immune plasmacytoid dendritic cells are present in JDM affected muscle (11) and may serve as a source of type I interferon (IFN) (12). These cells potentially play a role in JDM pathogenesis as many type I IFN inducible genes are significantly upregulated in JDM affected muscle (13), regardless of the duration of the inflammatory response prior to treatment (14).

NK cells (innate lymphocytes defined as CD56^+^ CD3^-^) have also been implicated in JDM pathogenesis. NK cells play a crucial role in the immune responses against viruses and transformed cells (e.g. tumors) by producing cytokines and secreting cytotoxic granules for targeted cell lysis (15–17). Several older studies present some evidence that NK cell mediated killing of target cells may be reduced in JDM patients (18, 19), and two previous studies have shown decreased peripheral NK cell percentages in JDM patients during active disease compared to controls (20, 21). We have also recently shown that treatment-naïve JDM patient NK cells have decreased calcium flux secondary to hypo-phosphorylation of phospholipase Cγ2 (PLCγ2), which substantially normalizes with treatment (20). However, it is still not well understood why NK cells are dysregulated in JDM and what implications this has for disease pathogenesis.

Here, we performed RNA sequencing (RNAseq) on sorted lymphocyte subsets from 4 new-onset JDM patients and healthy pediatric controls. The small cohort size is due to the rarity of this disease and treatment-naive samples; however, this cohort is of similar size to recently published JDM studies (22, 23) and other published JDM RNAseq studies (8). By sorting peripheral blood mononuclear cells (PBMCs) from JDM patients and controls into highly enriched T, B, and NK cell subsets prior to RNAseq, we were not only able to compare each cell subset between the patients and controls but were also able to compare amongst patient’s immune cell subsets as well. This approach confirmed previous published reports of increased type I interferon expression (24) and our prior report of the hyper-proliferative state of NK cells despite decreased overall NK cells in the JDM patients JDM (20). Furthermore, it provided novel insight into an enriched subset of NK cells with decreased ribosomal transcript expression in new-onset JDM patients. A similar subset of low ribosomal expressing NK cells in adult PBMCs from three healthy donors was recently identified using single-cell RNAseq (25), but was not further characterized or validated. We confirmed a small population of low ribosome expressing NK cells in PBMCs from healthy pediatric and adult controls and show this population to be substantially expanded in new-onset JDM NK cells. Furthermore, we demonstrate this increase in low ribosome NK cells is associated with decreased NK cell degranulation. We hypothesize this uniquely expanded population of low ribosome expressing NK cells may be playing a previously unappreciated role in JDM pathogenesis.

## Methods

### Patients

JDM was defined according to modified Bohan and Peter’s criteria (26). JDM patients diagnosed in the Pediatric Rheumatology clinics at St. Louis Children’s Hospital were eligible for enrollment if their cases were new-onset and treatment-naïve. The cohort of 4 patients used in the RNAseq studies was a subset of the 17 treatment naïve JDM patients from our previously published study delineating immune cell signaling defects in treatment-naïve JDM patients (20).

### Sample preparation for RNAseq

PBMCs from 4 new-onset JDM patients and 4 healthy pediatric controls were isolated using a Ficoll-Paque PLUS gradient (GE Healthcare) and cryopreserved. Immune cells were stained with CD56 (NCAM-1) BV421, CD3 (UCHT1) PerCP-Cy5.5, CD14 (M5E2) APC-Fire750, and CD19 (SJ25C1) FITC (Biolegend, San Diego, CA) and sorted to high purity using a FACSAria sorter (BD, Franklin Lakes, NJ). Live cells were sorted into three groups: NK cells (CD56^+^, CD3^-^, CD14^-^), B cells (CD19^+^, CD3^-^, CD14^-^), and T cells (CD3^+^, CD56^-^, CD14^-^) (**Supplemental Figure 2**). Purity ranges for patient subsets were 98.4 – 100% for NK cells, 96.1 – 99.1% for B cells, and 99.5 – 99.9% for T cells post sort. Purity ranges for healthy pediatric controls were 99.7 – 99.9% for NK cells, 95.1 – 98.1% for B cells, and 99.6 – 100% for T cells post sort. RNA was isolated with a RNeasy Micro Kit Plus (Qiagen, Germantown, MD). A cDNA library of mRNA was constructed using a Clontech SMARTer cDNA Synthesis Kit (Takara Bio USA, Mountain View, CA).

### RNAseq data acquisition, quality control, and processing

RNA sequencing was performed using a HiSeq3000 instrument (Illumina, San Diego, CA) with 50bp single-end reads, targeting 25-30 million reads per sample. Library construction and sequencing was performed by the Genome Technology Access Center (GTAC) at Washington University in St. Louis. RNAseq reads were aligned to the Ensembl release 76 top-level assembly with STAR version 2.0.4b (27). Gene counts were derived from the number of uniquely aligned unambiguous reads by Subread:featureCount version 1.4.5 (28). Transcript counts were determined by Sailfish version 0.6.3. Sequencing performance was assessed for total number of aligned reads, total number of uniquely aligned reads, genes and transcripts detected, ribosomal fraction known junction saturation, and read distribution over known gene models with RSeQC version 2.3 (29).

### qRT-PCR

Cryopreserved immune cells were thawed and stained with CD56 (NCAM-1) APC, CD3 (UCHT1) BUV395, and CD19 (SJ25C1) PE and sorted to high purity using a FACSAria sorter (BD, Franklin Lakes, NJ). Live cells were sorted into three groups: NK cells (CD56^+^, CD3^-^), B cells (CD19^+^, CD3^-^, CD56^-^), and T cells (CD3^+^, CD56^-^). RNA was isolated with a RNeasy Micro Kit Plus (Qiagen, Germantown, MD). A cDNA library of mRNA was constructed using SuperScript™ III Reverse Transcriptase Kit (Invitrogen, Waltham, MA). Samples were ran on an ABI One Step machine for the following markers: *RPS6, RPS27, RPS29, RPL13, MALAT1, and B2M* (30, 31). deltaCT (dCT) was determined by CT (*B2M)* – CT (Target).

### Degranulation assay

Cryopreserved immune cells were thawed and enriched for NK cells using the EasySep™ Human NK Cell Enrichment Kit (STEMCELL Technologies, Cambridge, MA). Enriched NK cells were cultured with or without K562 cells at a 1:1 concentration for 4 hours with Golgi Stop added after 1 hour. Cells were then prepared for flow cytometry with the following markers: Live/Dead Blue (Invitrogen, Waltham, MA), CD56 APC, CD18 APC-Cy7, CD16 BV605 (Biolegend, San Diego, CA), CD3 BUV395, CD7 BV711, CD107 PE (BD, Franklin Lakes, NJ), and IFITM1 FITC (Biorbyt, St. Louis, MO).

### pRPS6 flow cytometry staining

Cryopreserved immune cells were thawed and enriched for NK cells using the EasySep™ Human NK Cell Enrichment Kit (STEMCELL Technologies, Cambridge, MA). Enriched NK cells were stained with the following surface markers for flow cytometry: Live/Dead Blue, CD56 APC, CD3 BUV395, CD19 BV510 (BD, Franklin Lakes, NJ), CD18 APC-Cy7, CD7 BV711, CD16 BV605, and IFITM1 FITC. Cells were then stimulated with the following for 15 min: 500U/ml IL-2, 50ng/ml IL-12, 500ng/ml LPS, 500U/ml IFNα_a4_, and 1ug/ml α-mouse IgG. After cytokine stimulation, cells were fixed with 4% paraformaldehyde for 10 min and then permeabilized with methanol for 30 min. Samples were then stained with the following intracellular antibodies overnight: pRPS6 BV421 (Biolegend, San Diego, CA). Samples were ran on a 5-laser Aurora (Cytek Biosciences, Fremont, CA).

### Statistics

All gene counts were imported into the R/Bioconductor package EdgeR (32) and trimmed mean of M values (TMM) normalization size factors were calculated to adjust for samples with differences in library size. Ribosomal genes and genes not expressed in the smallest group size minus one sample greater than one count-per-million were excluded from further analysis. The TMM size factors and the matrix of counts were then imported into the R/Bioconductor package Limma (33). Weighted likelihoods based on the observed mean-variance relationship of every gene and sample were then calculated for all samples with the voomWithQualityWeights (34). The performance of all genes was assessed with plots of the residual standard deviation of every gene to their average log-count with a robustly fitted trend line of the residuals. Differential expression analysis was then performed to analyze for differences between conditions and the results were fitted for only those genes with Benjamini-Hochberg false-discovery rate adjusted P values less than or equal to 0.05.

For each contrast extracted with Limma, global perturbations in known Gene Ontology (GO) terms and KEGG pathways were detected using the R/Bioconductor package GAGE (35) to test for changes in expression of the 2 log fold-changes reported by Limma in each term versus the background log 2 fold-changes of all genes found outside the respective term. The R/Bioconductor package heatmap3 (36) and Pathview (37) were used to display heatmaps or annotated KEGG graphs across groups of samples for each GO term or KEGG pathway, respectively, with a Benjamini-Hochberg false-discovery rate adjusted P value less than or equal to 0.05.

To find the most critical genes, the raw counts were variance stabilized with the R/Bioconductor package DESeq2 (38) and then analyzed *via* weighted gene correlation network analysis with the R/Bioconductor package WGCNA (39). Briefly, all genes were correlated across each other by Pearson correlations and clustered by expression similarity into unsigned modules using a power threshold empirically determined from the data. An eigengene was then created for each *de novo* cluster, and its expression profile was correlated across all coefficients of the model matrix. These *de-novo* clustered genes were then tested for functional enrichment of known GO terms with hypergeometric tests available in the R/Bioconductor package clusterProfiler (40). Significant terms with Benjamini-Hochberg adjusted P values less than 0.05 were then collapsed by similarity into clusterProfiler category network plots to display the most significant terms for each module of hub genes in order to interpolate the function of each significant module. The information for all clustered genes for each module were then combined with their respective statistical significance results from Limma to determine whether those features were also found to be significantly differentially expressed.

Validation experiments for the RNAseq results (**Figure 4B**) were done with paired treatment naïve JDM patients and AMC (n = 6 pairs) in triplicate on individual days (**Figure 4**). Verification studies of the low ribosome NK population (**Figures 5**–**7**) were similarly performed with paired samples in triplicate with n = 4-6 paired samples, each run on individual days (with paired conventional NK cells and sorted low ribosomal NK from healthy adult controls in **Figure 5**, paired NK cells from treatment naïve JDM patients and AMC in **Figure 6**, and paired NK Cells from clinically inactive JDM patients and AMC in **Figure 7**). Experiments with the paired samples were performed on the same day with 3 replicates in each assay. Data consisting of two groups used a two-tailed paired *t* test. Significance levels were set at *P <*0.05.

### Study approval

The study was approved by the institutional review boards at Washington University School of Medicine, St. Louis (IRB ID 201109216), and written informed consent was received to use patient samples prior to participation.

## Results

### JDM patients and healthy controls

Average age of the 10 JDM patients and 10 healthy pediatric controls (HPC) used in these studies were 9.5 and 10.35, respectively with similar sex distributions between each group (**Table 1**). The average age of the 8 new onset JDM patients and 8 healthy controls were respectively 9 and 10, and the average age for the 4 new onset JDM patients and controls used in the RNAseq study was 9.1 and 12.1 years, respectively. The duration of untreated disease in JDM patients was similar in the RNAseq cohort (2.75 months average with a standard deviation of 1.25 months). Nine of the ten JDM patients used in the study were positive for a myositis-specific autoantibody (MSA). All ten JDM patients were negative for myositis-associated autoantibodies (MAA). Six treatment-naïve JDM patients and 6 age-matched controls (AMCs) (including 1 JDM patient and AMC from the RNAseq cohort) were used in additional validation experiments. The average age of the 6 JDM patients used for validation experiments was 7.95 years and 8.76 years for the AMCs with 3 males and 3 females in each group. Four patients (two of whom also had treatment naïve samples in the study) with clinically inactive disease (CID) on medication were used in the follow-up studies. Patient presentation and medicine regimen (for JDM patients with clinically inactive disease) is listed in **Table S1**. Three of the four JDM patients used in the RNAseq studies were treatment naïve. The fourth JDM patient had new-onset JDM and was initially thought to also be treatment naïve. However, after the RNAseq studies were completed, we determined that patient 3 had given informed consent during his initial hospitalization prior to starting therapy but that his initial research blood sample was not drawn until his first clinic visit 5 weeks later. At that time, he had received methylprednisolone (2mg/kg i.v. for 3 days) followed by oral prednisone (0.8 mg/kg/day titrated down to 0.4 mg/kg/day over 5 weeks) and subcutaneous methotrexate (12 mg/m^2^/wk). In our previous CyTOF study (20), patient 3’s PLCy2 hypo-phosphorylation was no different than that of the 16 treatment-naïve JDM patients, demonstrating that early therapy with corticosteroids and methotrexate was not sufficient to attenuate the observed NK cell PLCy2 dysregulation in JDM. Additionally, the results and overall conclusion of the RNAseq studies were essentially unchanged with the exclusion of patient 3 in a sub-analysis (**Figure S1**) and still clearly highlighted the low NK cell ribosome levels in JDM patients PBMCs compared to healthy controls.

**Table 1:** Patient demographics and characteristics.

JDM Patient	Sex	Race	Duration of untreated disease at diagnosis (months)	Age at sample collection (yrs)	MSA	Treatment Naive	RNAseq	Validation Experiments	Clinically Inactive JDM Experiments
1	M	C	3	3.2	negative	*	X	X	
2	F	C	<3	8.2	p155/140	*	X		
2b	F	C		14.2					X
3	M	C	4	12.1	MDA5		X		
4	F	C	<1	12.8	MJ	*	X	X	
5	M	C	4	10.7	p155/140	*		X	
6	F	C	1	7.9	MDA5	*		X	
6b	F	C		9.8					X
7	F	C	<1	14.5	Mi-2	*		X	
8	M	C	<1	2.6	MJ	*		X	
**JDM PT CID**	**Sex**	**Race**							
9	F	C	2	10.3					X
10	F	C	1.5	8.7					X
**HPC**									
1	M	C		7.5			X	X	
2	F	C		9.4			X		
3	F	C		13.4			X	X	
4	F	C		18.3			X		
5	M	C		10.1				X	
6	F	C		8.2				X	
7	F	C		10.2				X	
8	M	C		3.2				X	
9	F	C		9					X
10	F	C		10					X
11	F	C		11					X
12	F	C		14					X

Average age of the 10 JDM patients and 10 healthy pediatric controls (HPC) used in these studies were 9.58 and 10.35, respectively with similar sex distributions between each group. The average age of the 8 new onset JDM patients and 8 heathly controls were respectively 9 and 10.03, and the average age for the 4 new onset JDM patients used in the RNAseq study was 9.05 and 12.15 years, respectively, The duration of untreated disease in JDM patients was similar in the RNAseq cohort (2.75 months average with a standard deviation of 1.25 months). All ten JDM patients were negative for myositis-associated autoantibodies (MAA). Six treatment-naïve JDM patients and 6 age-matched controls (AMCs) (including 1 JDM patient and AMC from the RNAseq cohort) were used in additional validation experiments. The average age of the 6 JDM patients used for validation experiments was 7.95 years and 8.76 years for the AMCs with 3 males and 3 females in each group. Four patients (two of whom also had treatment naïve samples in the study) with clinically inactive disease (CID) on medication were used in the follow-up studies. Other abbreviations: C (Caucasian), MSA (myositis-specific antibodies), MDA5 (anti melanoma differentiation associated gene 5), an MJ also known as nuclear matrix protein 2 (NXP-1), and Mi2 and p155/140 are also MSAs.

### Differentially expressed genes in NK cells, B cells, and T cells

**Figure 1:**
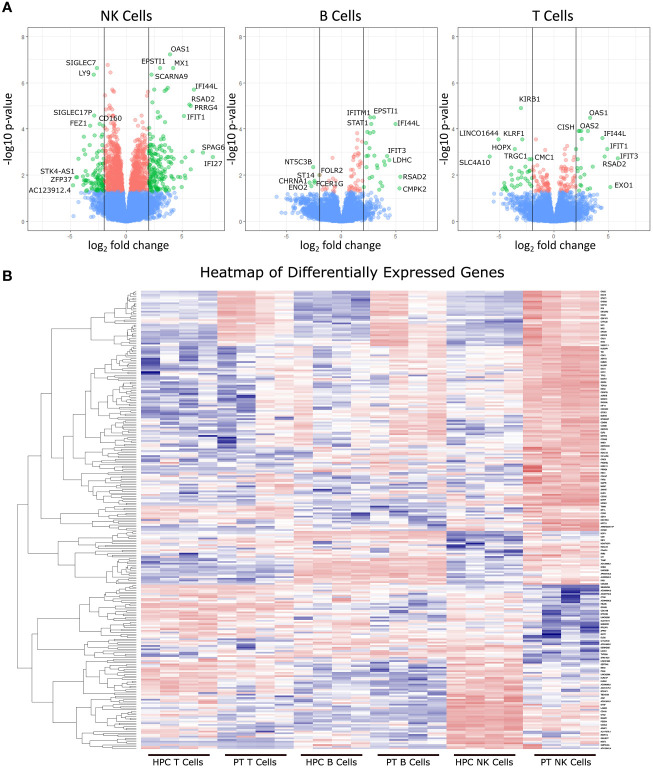
Differentially expressed genes from JDM new-onset patients compared to healthy pediatric controls. Gene expression in JDM new-onset patients was compared to gene expression in healthy pediatric controls for each cell type to calculate the log fold-change (logFC) and adjusted P value for each gene. Differentially expressed genes were classified as having a greater than 2-log fold change (either positive or negative) and having an adjusted P value of < 0.05 **(A)**. The top three panels depict volcano plots of differentially expressed genes from each cell type comparing JDM patients (PT) to age-matched controls (AMC). Green dots represent differentially expressed genes with an adjusted P value of < 0.05 and a greater than 2-log change in expression. Red dots represent genes with an adjusted P value of < 0.05 but a less than 2-log fold change in expression, and blue dots represent genes with an adjusted P value > 0.05. The bottom panel **(B)** shows a heatmap of differentially expressed genes (adjusted P value of < 0.05 and a greater than 2-log change in expression) in JDM patient NK cell, T, and B cells compared to immune cells from controls with red and blue representing upregulation and downregulation, respectively.

**Figure 2:**
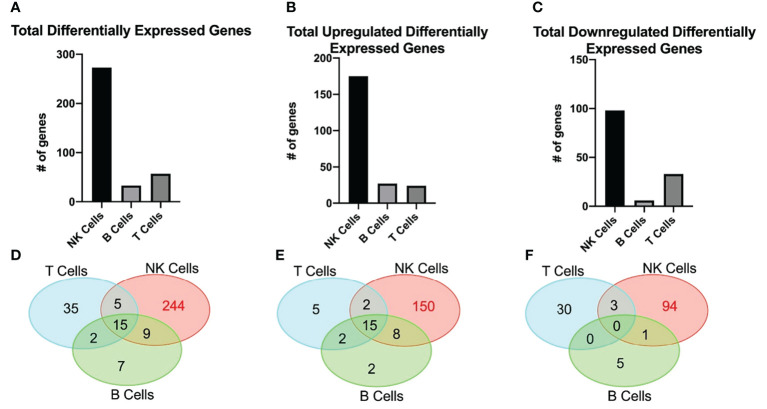
JDM new-onset patients have a greater number of both upregulated and downregulated differentially expressed genes in NK cells than in T or B cells. The number of total **(A)**, upregulated **(B)**, and downregulated **(C)** differentially expressed genes in NK, B, and T cells is shown. The number of differentially expressed genes that overlap among NK, B, and T cell is shown by either total **(D)**, upregulated **(E)**, or downregulated **(F)** by Venn Diagram. Differentially expressed genes were classified as having a greater than 2-log fold change (either positive or negative) and having an adjusted P value of < 0.05.

RNAseq was used to determine differentially expressed genes between new-onset JDM patients and healthy pediatric controls, using highly enriched NK (98.3%), T (99.7%), and B (96.6%) cells sorted from live PBMCs (**Figure 1**). We chose to focus on these lymphoid subsets based on our previously published CyTOF data in JDM, showing that the only significant differences in the percentages of 23 leukocyte subsets in samples from 17 treatment-naïve JDM patients and controls were in NK, T, and B cells (20). Sorting cells prior to analysis allowed for comparisons not only between JDM patients and controls but also among the lymphocyte populations isolated from JDM patients and controls. We first compared the number of differentially expressed genes among each cell type. This was done by comparing the gene expression between the JDM patients and controls for each immune subset. Differentially expressed genes were defined as having a greater than 2-log fold change (logFC), either positive or negative, as well as having an adjusted P value of < 0.05 (corrected for multiple hypothesis testing). NK cells from new-onset JDM patients had a greater number of differentially expressed genes compared to controls than either B or T cells (**Figure 1A**). As shown in the heatmap in **Figure 1B**, the JDM PT NK cells had a distinct set of upregulated (red) and downregulated (blue) genes compared with the other cell types. When the number of differentially expressed genes was quantified, NK cells had a substantially greater number of total (**Figure 2A**), upregulated (**Figure 2B**), and downregulated (**Figure 2C**) differentially expressed genes compared to B or T cells. Additionally, there was only modest overlap in differentially expressed genes among each cell type (**Figure 2D**), other than in upregulated genes related to cell proliferation and viral immune response (**Figure 2E** and **Table S2**). In the downregulated differentially expressed genes, there was little to no overlap (**Figure 2F** and **Table S2**), suggesting each cell type experiences unique gene downregulation in JDM. Together, these data show that NK cells have a greater number of both upregulated and downregulated differentially expressed genes compared to B and T cells, with minimal overlap amongst these three lymphocyte populations, demonstrating that gene expression in NK cells is more highly altered in new-onset JDM patients than what is observed in T and B cells.

### Gene ontology analysis in the three lymphocyte subsets

Given that NK cells have a substantially greater number of differentially expressed genes, we chose to focus further analysis primarily on NK cells. We next wanted to see which biological process Gene Ontology (GO) groups were up or downregulated in new-onset JDM NK cells compared to controls. The upregulated groups for JDM patients were primarily related to either cell proliferation or innate immune viral response for biological process GO categories (**Table 2**). This supports previous findings of type I IFN upregulation in JDM patients (24). We have previously shown that treatment-naïve JDM patient NK cells are highly activated and proliferative as measured by CD69 and Ki67, respectively (20), and our current RNAseq analysis confirms this finding. B and T cells also had significant upregulation of cell activation and immune response genes (**Supplemental Tables 3** and **4**), as well as increased type I IFN response genes, confirming previously published data of an increased type I IFN response genes in JDM treatment-naïve B cells (8). These findings suggest that while all three lymphocyte subsets are activated and have upregulated type I IFN in JDM, NK cells are uniquely highly proliferative as well.

**Table 2:** Top upregulated and downregulated NK cell Gene Ontology (GO) values in JDM patients.

Accession	Description	Number of Genes	logFC	P value
GO:1903047	Mitotic cell cycle process	712	7.351245	1.66E-13
GO:0000278	Mitotic cell cycle	818	7.317412	1.99E-13
GO:0022402	Cell cycle process	1065	6.939867	2.60E-12
GO:0007049	Cell cycle	1425	6.906268	3.06E-12
GO:0006950	Response to stress	2661	6.84279	4.32E-12
GO:0051276	Chromosome organization	991	6.797868	7.00E-12
GO:0006952	Defense response	1004	6.662913	1.75E-11
GO:0002376	Immune system process	2034	6.383504	9.63E-11
GO:0007059	Chromosome segregation	265	6.456295	1.26E-10
GO:0044772	Mitotic cell cycle phase transition	478	6.18707	4.57E-10
GO:0043207	Response to external biotic stimulus	574	6.143942	5.66E-10
GO:0051707	Response to other organism	574	6.143942	5.66E-10
GO:0000819	Sister chromatid segregation	166	6.275086	5.76E-10
GO:0006955	Immune response	1420	6.034535	9.02E-10
GO:0044770	Cell cycle phase transition	507	6.060856	9.58E-10
GO:0009607	Response to biotic stimulus	601	6.048269	9.94E-10
GO:0045087	Innate immune response	619	6.044385	1.01E-09
GO:0009605	Response to external stimulus	1348	5.91724	1.85E-09
GO:0098813	Nuclear chromosome segregation	212	6.021872	1.95E-09
GO:0006260	DNA replication	243	5.881377	3.80E-09
GO:0006259	DNA metabolic process	833	5.762369	4.94E-09
GO:0098542	Defense response to other organism	299	5.814659	5.30E-09
GO:0048285	Organelle fission	337	5.766276	6.33E-09
GO:0051301	Cell division	479	5.727649	6.89E-09
GO:0000280	Nuclear division	305	5.709448	9.05E-09
GO:0006613	Co-translational protein targeting to membrane	97	-4.02271	4.54E-05
GO:0006614	SRP-dependent co-translational protein targeting to membrane	93	-4.02124	4.64E-05
GO:0045047	Protein targeting to ER	106	-3.87484	7.71E-05
GO:0072599	Establishment of protein localization to endoplasmic reticulum	109	-3.74763	0.000122

List of the top 25 significantly upregulated and the 4 significantly downregulated Biological Process GO categories in new-onset JDM NK cells compared to healthy pediatric controls.

We next looked at the downregulated biological process GO groups. New-onset JDM NK cells displayed a significant downregulation in groups relating to protein targeting to the endoplasmic reticulum (ER) (**Table 2**). Interestingly, the downregulated GO values in both B and T cells did not include any genes related to protein targeting to the ER (**Supplemental Tables 3** and **4**) suggesting that in JDM this downregulation of gene expression in protein targeting to ER is specific to NK cells among lymphocytes.

### Weighted correlation network analysis in T, B, and NK cells

To further confirm our results, we utilized a weighted correlation network analysis (WGCNA), which is used to find clusters of highly correlated genes and compared them among different experimental groups. Looking at the trait and significance chart in **Table 3**, the module that was most significantly upregulated with the highest P value in our new-onset JDM patients compared to all other groups was Module 7 (Spearman Correlation Coefficient = 0.788, and P value = 0.0000047). This module was not significantly correlated in T or B cells from JDM patients compared to controls (**Table 3**). As with the previous GO analysis (**Table 2**), the genes with the highest correlation module membership, which are representative of the theme of the entire module, are related to cell proliferation and activation (**Supplemental Table 5**), again confirming that NK cells from JDM patients are highly activated and highly proliferative compared to both control NK cells and other lymphocytes in JDM patients.

**Table 3:** WGCNA analysis of JDM NK Cells.

Module	HPC NK Cell	HPC B Cell	HPC T Cell	PT NK Cell	PT B Cell	PT T Cell
1	-0.446 0.0058	0.338 0.11	0.337 0.11	-0.69 0.00019	0.255 0.23	0.305 0.15
2	0.669 0.00036	-0.385 0.064	-0.126 0.56	0.547 0.0057	-0.444 0.03	-0.261 0.22
3	-0.108 0.62	-0.076 0.72	-0.147 0.49	0.043 0.84	0.124 0.56	0.164 0.44
4	0.284 0.18	-0.581 0.0029	0.442 0.031	0.054 0.8	-0.627 0.001	0.428 0.037
5	-0.382 0.066	-0.45 0.028	-0.396 0.055	0.59 0.0024	0.321 0.13	0.316 0.13
6	0.098 0.65	-0.544 0.006	-0.094 0.66	0.631 0.00094	-0.235 0.27	0.143 0.5
7	-0.062 0.78	-0.101 0.64	-0.495 0.014	0.788 0.0000047	0.199 0.35	-0.33 0.12
8	0.192 0.37	0.105 0.63	-0.082 0.7	-0.215 0.31	-0.04 0.85	0.04 0.85
9	0.532 0.0075	-0.18 0.4	0.077 0.72	0.152 0.48	-0.213 0.32	-0.368 0.077
10	0.15 0.48	0.422 0.04	-0.596 0.0021	0.225 0.29	0.424 0.039	-0.625 0.0011
11	-0.265 0.21	-0.224 0.29	0.63 0.00097	-0.445 0.029	-0.307 0.14	0.611 0.0015
12	-0.335 0.11	0.636 0.00083	-0.345 0.098	-0.28 0.18	0.621 0.0012	-0.296 0.16
13	-0.139 0.52	0.345 0.098	-0.33 0.12	0.426 0.038	0.06 0.78	-0.362 0.082

Module trait and significance chart from the WGCNA (weighted correlation network analysis). The top value for each module is the Spearman Correlation Coefficient and the bottom value is the P value. Color intensity representing the correlation coefficient value with blue indicating a negative correlation, while orange indicates a positive correlation. The genes with the highest correlation coefficient are shown in **Table S4** for Module 7 and **Table S5** for Module 1.

We also examined modules that were negatively correlated with the JDM patient NK cells and significantly different than all other experimental groups. Module 1 fits these criteria with a Spearman Correlation Coefficient of -0.69 and P value of 0.00019 (**Table 3**). The genes with the highest correlation module membership in Module 1 involve ribosomes and ER signaling (**Supplemental Table 6**), consistent with the previous GO analysis (**Table 2**). NK cells have significantly more ribosomes at baseline than other lymphocytes (41), and therefore defects pertaining to ribosomes will likely be most noticeable in NK cells. This further demonstrates ribosomes and subsequent ER function are downregulated in JDM patient NK cells.

### Generally applicable gene-set enrichment and KEGG analysis of NK cells

**Figure 3:**
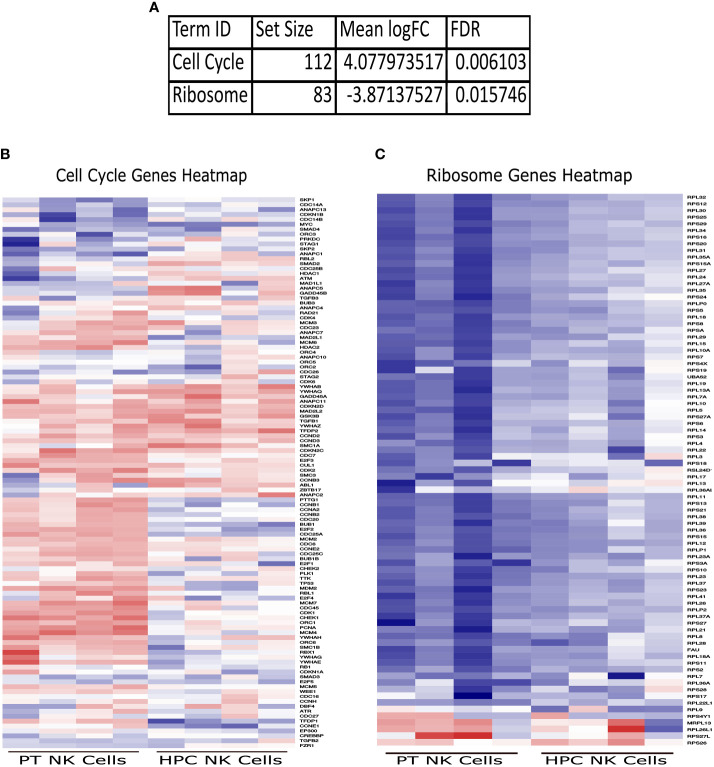
Significantly upregulated and downregulated GAGE KEGG Canonical Pathway gene sets. **(A)** Set size, logFC, and FDR for each significantly different gene set. Heatmaps of genes within the Cell Cycle **(B)** and Ribosome **(C)** gene sets in JDM patient NK cells and healthy pediatric control NK cells. Red indicates upregulation, while blue indicates downregulation.

To further our understanding of the data, we performed a GAGE (Generally Applicable Gene-set Enrichment) (35) analysis and utilized the Molecular Signatures Database (MSigDB) to determine any gene sets significantly different in the JDM patient NK cells. While there was no change in most of the gene sets, there were two gene sets in the KEGG (Kyoto Encyclopedia of Genes and Genomes) subset analysis of the Canonical Pathways that were significantly different in JDM patient NK cells compared to controls (**Figure 3A**). The Cell Cycle gene set was significantly upregulated (**Figure 3B**), while the Ribosome gene set was significantly downregulated (**Figure 3C**). This confirmed the previous WGCNA analysis of a significant increase of genes involved with cell cycle and a significant decrease of genes involved with ribosomal signaling in the JDM NK cells (**Table 3**).

**Figure 4:**
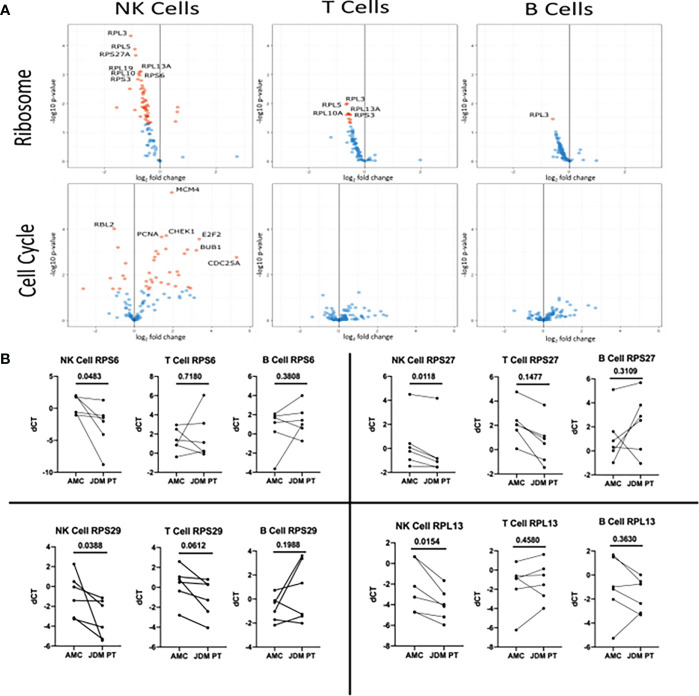
Volcano plot of genes in the KEGG Ribosome and KEGG Cell Cycle sets in new-onset JDM cells compared to healthy pediatric controls and qRT-PCR validation of select Ribosome genes in treatment-naïve JDM patients vs age-matched controls (AMC). **(A)** Genes in the KEGG Ribosome (hsa03010) and Cell Cycle pathway (hsa04110) in JDM NK, T, and B cells compared to AMC. The Ribosome pathway has many ribosomal proteins while the Cell Cycle pathway contains genes involved in regulation of cell proliferation (listed in **Table S6**). Red dots indicate an adjusted P value of < 0.05 and blue dots indicate an adjusted P value of > 0.05. **(B)** qRT-PCR conformation of select genes in the KEGG Ribosome (hsa03010) pathway in JDM NK, T, and B cells compared to pediatric controls. deltaCT (dCT) was determined by CT of *B2M* – target CT.

We next wanted to see which ribosomal and cell cycle associated component’s gene expression was differentially modulated in new-onset JDM patient NK cells compared to healthy pediatric controls. To do this, we utilized the KEGG PATHWAY database, which contains already determined gene sets and graphical representations of those GAGE gene sets (42). By utilizing the Cell Cycle (hsa04110) and Ribosome (hsa03010) reference pathways (**Supplemental Table 7**), we were able to visualize log fold change of genes involved in the regulation of the cell cycle and ribosomes genes. As shown in **Figure 4A**, most of the cell cycle associated genes were upregulated while the ribosomal protein associated genes were downregulated in JDM patient NK cells compared to controls, although only a subset of the genes met the criteria to be denoted significantly different after multiple hypothesis correction. (See marked list in **Table S7** of genes that were statistically significant after accounting for multiple hypothesis correction.) Additionally, we confirmed that select ribosomal genes had decreased transcription as measured by qRT-PCR (**Figure 4B**) from sorted NK, T, and B cells from new-onset JDM patients compared to healthy pediatric controls. This further supports the observation that NK cell ribosome biogenesis is downregulated, potentially due to the prolonged, highly active state of these NK cells in new-onset JDM patients.

### Conformation of low ribosome expressing NK cell population

**Figure 5:**
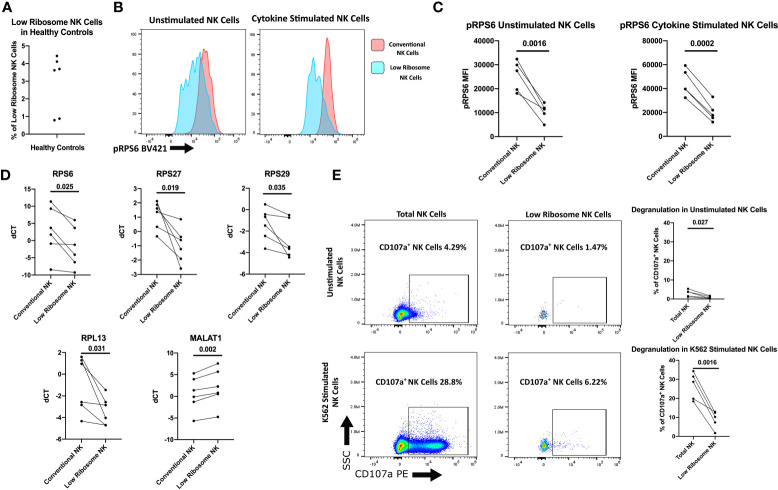
Characterization of the low ribosome expressing NK cell population. Healthy adult donors were used in these experiments. **(A)** Percentage of low-ribosome expressing NK cells in the total NK cell population measured by flow cytometry with the following markers: CD56^+^, CD3^-^, CD18^+^, CD7^-^, CD16^low^, and IFITM1^-^. **(B)** Representative histogram of pRPS6 as measured by flow cytometry in low ribosome population compared to the conventional ribosome population: CD56^+^, CD3^-^, CD18^+^, CD7^+^, and CD16^high^. **(C)** pRPS6 in unstimulated and IL-2, IL-12, LPS, IFNα_A4_, CD16 cross-linking stimulated NK cells in the low ribosome expressing NK population and the conventional NK population. **(D)** qRT-PCR measurement of ribosomal targets and *MALAT1* in sorted populations of low ribosome expressing NK cells and conventional NK cells. dCT was determined by CT of *B2M* – target CT. **(E)** Representative flow cytometry plot of CD107a degranulation measured in unstimulated NK cells and NK cells stimulated with target cells in the total NK cell population and low ribosome expressing NK cell population. CD107a^+^ NK cells in unstimulated and K562 stimulated NK cells of the total NK cell population and the low ribosome expressing NK cell population.

A recently published study utilizing single-cell RNAseq (scRNAseq) of NK cells from three healthy adult donors reported a population of NK cells which are highly activated and have low ribosome transcription (25). Although these low ribosome expressing NK cells comprised less than 2% of the total NK population in the adult controls, we hypothesized this population may be expanded in NK cells from treatment naïve JDM patients, contributing to the decreased ribosomal RNA in the total NK cell population. Additionally, we hypothesize these cells have decreased NK functionality compared to the rest of the NK cell population. First, we wanted to confirm the existence of this NK cell population in healthy adult NK cells and determine if there were functional differences in this population compared to the total NK population. We were able to determine surface markers to identify the low ribosome population from the scRNAseq study transcription data without the need for ribosome markers, which would require permeabilizing the cell for intracellular staining, rendering the cells unable to be used for functional assays. The following markers were used to identify this population of low ribosome expressing cells: CD56^+^, CD3^-^, CD18^+^, CD7^-^, CD16^low^, and IFITM1^-^ (25). Using flow cytometry, we were able to confirm that the low ribosome NK cell population made up a small percentage of the total NK cell population (**Figure 5A**) in healthy adults, confirming the transcription data shown previously. This population was transcriptionally similar to another larger cluster of NK cells that were highly activated but did not have decreased ribosomal expression: CD56^+^, CD3^-^, CD18^+^, CD7^+^, and CD16^high^. These NK cells were used as a representative for the conventional NK cell population when determining levels of RPS6 phosphorylation. The full gating strategies for both populations are shown in **Figure S3B**. As shown in **Figures 5B, C**, the low ribosome expressing population had decreased RPS6 phosphorylation compared to the conventional NK cell population as determined by flow cytometry in unstimulated NK cells (gating strategy shown in **Figure S3C**) and also in NK cells stimulated with IL-2, IL-12, LPS, IFNα_A4_, and CD16 cross-linking for 15 minutes. Because RPS6, a component of the 40s ribosomal subunit, is involved in translation and its phosphorylation leads to the production of additional ribosomal proteins, it is often used as a measurement of pan-ribosomal synthesis, mTOR signaling, and overall translation potential of the cell (43). To further confirm this population has decreased ribosomal RNA levels, qRT-PCR was performed on sorted low ribosome and conventional NK cells for the following ribosomal targets: *RPS6, RPS27, RPS29*, and *RPL13*. All these ribosomal targets were significantly downregulated in our RNAseq data and were shown to have decreased levels in the low ribosome expressing NK cell subset in healthy adult donors (**Figure 5D**). We further confirmed this low ribosome expressing NK population was the same population identified by scRNAseq by assessing the upregulation of *MALAT1* levels, a uniquely upregulated gene identified only in the low ribosome expressing NK cell cluster in the transcript data (25). *MALAT1* transcription was significantly increased in our low ribosome population compared to the conventional population (**Figure 5D**), confirming our ability to utilize surface markers to identify this population of low ribosome expressing NK cells. Now that we had demonstrated the ability to identify the low ribosome expressing NK cells, we wanted to determine if this population had decreased NK cell function as assessed *via* degranulation when exposed to target cells, an essential function of NK cells. As shown in **Figure 5E**, the low ribosome expressing NK cells have significantly decreased NK cell degranulation after stimulation, as measured by CD107a, compared to the total NK cell population (full gating strategy shown in **Figure S3D**). This set of experiments confirms the presence of a low ribosome expressing population of NK cells at a low percentage in healthy adult PBMCs and shows an associated decreased NK cell function of this population for the first time.

### Low ribosome expressing NK cell population in JDM patients

**Figure 6:**
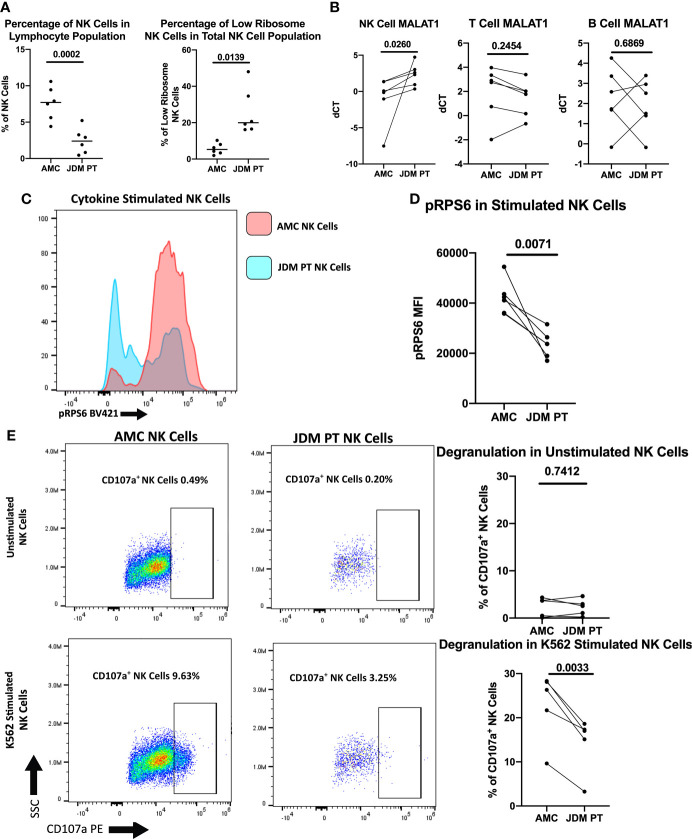
Comparison of NK cells of treatment-naïve JDM patients and age-matched controls. **(A)** Percentages of NK cells in total PBMCs and low ribosome expressing NK cells in the NK population in JDM PT and AMC. **(B)** qRT-PCR measurement of *MALAT1* in sorted populations of NK, T, and B cells in treatment-naïve JDM PT compared to AMC. dCT was determined by *B2M* CT – *MALAT1* CT. Representative histogram **(C)** and MFI **(D)** of pRPS6 as measured by flow cytometry in stimulated, enriched NK cells from treatment-naïve JDM PT and AMC. **(E)** NK Cell degranulation of treatment-naïve JDM PT and AMC in enriched NK cells unstimulated or stimulated with K562 cells. A representative flow plot is shown.

Next, we investigated if this population of dysregulated NK cells was expanded in treatment-naïve JDM patients, potentially contributing to decreased NK cell function. Although JDM patients have an overall decreased percentage of peripheral NK cells, the low ribosome expressing NK cells comprised a substantially greater percentage of the total NK cell population in the JDM patients compared to healthy pediatric controls (**Figure 6A**). Representative flow plots are shown in **Figures S4A, B**. We have shown decreased RNA levels of ribosomal targets in treatment-naïve NK cells (**Figure 4B**). Additionally *MALAT1*, shown to be uniquely upregulated in low ribosome NK cells (25), was significantly upregulated in JDM NK cells compared to healthy controls (**Figure 6B**). We also measured RPS6 phosphorylation by flow cytometry and showed treatment-naïve JDM NK cells have decreased RPS6 phosphorylation compared to AMC (**Figures 6C, D**). We next wanted to determine if the increased population of low-ribosome expressing NK cell in JDM patients alters NK cell function. As shown in **Figure 6E**, JDM patient NK cells have decreased degranulation compared to healthy pediatric controls (full gating strategy shown in **Figure S4C**). Overall, this shows the expansion of a newly defined population of low-ribosome expressing NK cells in JDM treatment-naïve patients and implicates this expanded low ribosomal population in contributing to decreased NK cell function as assessed here by decreased NK cell degranulation.

### Low ribosome expressing NK cell population in JDM patients with clinically inactive disease

**Figure 7:**
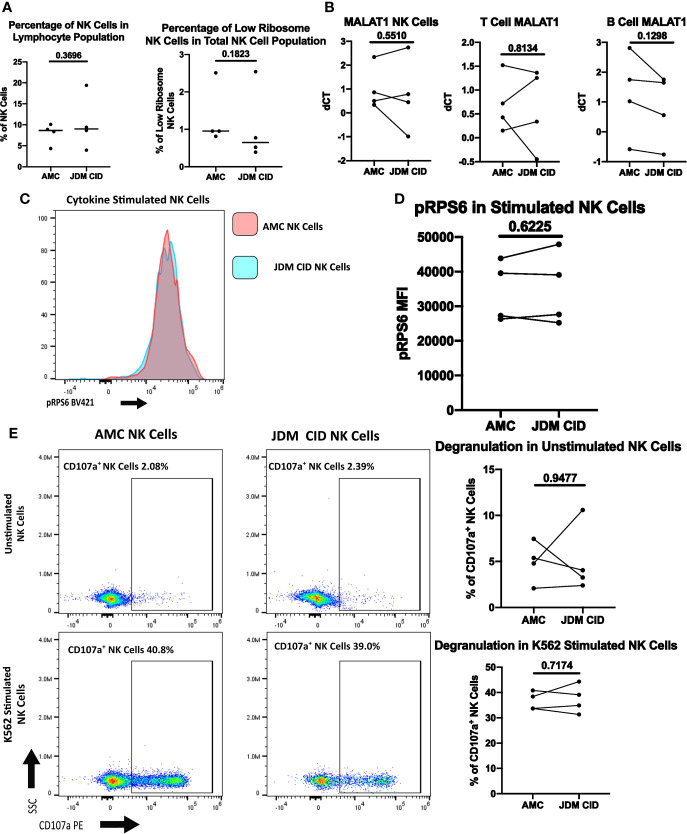
Comparison of NK cells of clinically inactive JDM patients and age-matched controls. **(A)** Percentages of NK cells in total PBMCs and low ribosome expressing NK cells in the NK population in JDM PT with clinically inactive disease (CID) and AMC. **(B)** qRT-PCR measurement of *MALAT1* in sorted populations of NK, T, and B cells in JDM CID PT compared to AMC. dCT was determined by *B2M* CT – *MALAT1* CT. Representative histogram **(C)** and MFI **(D)** of pRPS6 as measured by flow cytometry in stimulated, enriched NK cells from JDM CID PT and AMC. **(E)** NK Cell degranulation of JDM CID PT and AMC in enriched NK cells unstimulated or stimulated with K562 cells. A representative flow plot is shown.

We next wanted to determine if JDM patients would continue to have an expanded low ribosome expressing NK cell population after achieving clinically inactive disease (CID) on treatment. Four JDM patients that had achieved CID on treatment were used in these studies, including two patients whose treatment-naïve samples were used in previous experiments. As shown in **Figure 7A**, both the percentage of NK cells and the percentage of low ribosome expressing NK cells normalize compared to the age-matched controls in JDM patients with CID. In **Figure 7A**, patient 10 had been treated with a two dose course of rituximab shortly after diagnosis (approximately 7 month prior), altering their percentage of NK cells (19.4%) due to the low percentage of B cells but not impacting the percentage of low ribosome expressing NK cells (0.77%). Additionally, the transcription of *MALAT1*, uniquely upregulated in low ribosome expressing NK cells, was similar in NK, T, and B cells in age-matched controls and JDM CID (**Figure 7B**). Phosphorylation of RPS6, an indicator of pan-ribosomal synthesis, was no different in stimulated NK cells in JDM CID and AMC (**Figures 7C, D**). Degranulation of JDM NK cells also recovered with treatment (**Figure 7E**) with no difference compared to the AMC. Additionally, we were able to run PT 6 pre-treatment and clinically inactive disease samples simultaneously. Shown in **Figure S5**, all values measured were recovered upon the JDM patient reaching clinically inactive disease. This set of experiments demonstrates that JDM patients in clinical remission on treatment regain their NK cell percentages and function and lose their expansion of the low ribosome expressing NK cell population.

## Discussion

NK cells have been implicated in the pathogenesis of a number of autoimmune diseases, including systemic lupus erythematosus (SLE), multiple sclerosis (MS), and rheumatoid arthritis (RA) (44, 45). More recently, we reported decreased peripheral NK cells in treatment-naïve JDM patients with suppressed NK cell PLCγ2 phosphorylation that correlated with decreased calcium flux (20), potentially providing a mechanism to account for decreased NK cell killing observed in JDM patients in several small studies (18, 19).

NK cells are innate lymphocytes that play an immune regulatory role through the generation of immunomodulatory cytokines and chemokines and by directly killing infected, transformed, or dysregulated cells (17). Although prior studies have highlighted the potential role of several immune cell subsets in JDM pathogenesis [e.g. B cells (8) and plasmacytoid dendritic cells (pDCs) (46)], the etiology of JDM remains poorly characterized. Here, we present RNAseq analysis of highly enriched lymphocyte subsets from new-onset JDM patients that further implicates NK cell dysregulation in potentially contributing to JDM pathogenesis. Accumulating evidence has implicated NK cell-mediated regulation of T cells in contributing to the suppression of autoreactive T cells (47–50). Unpublished data from our previous JDM CyTOF study (20) shows a significant reduction in JDM T cell and NK cell activation and proliferation (as measured by CD69 and Ki-67, respectively) upon reaching clinical remission (**Supplemental Figure 6**). We hypothesize NK cell dysregulation may eliminate a check on auto-reactive T cells (17, 51), potentially contributing to the onset of JDM, and that this normalizes after achievement of inactive disease with treatment. While the specific role of NK cells in suppressing autoreactive T cells has not yet been directly investigated in JDM, suppressed NK cell cytotoxicity against T cells has been described in Multiple Sclerosis (MS) (52–54).

This is the first study to utilize RNAseq on multiple sorted immune cell subsets to compare differential upregulated and downregulated gene expression in NK, B, and T cells from new-onset JDM patients and healthy pediatric controls. The only four prior published RNAseq studies in JDM that we are aware of include a study of total PBMCs from JDM patients (55), plasma exomes from JDM patients (56), study of B cells of JDM patients (8), and a comparison of JDM treatment responders vs non-responders (57). While these studies have mainly explored JDM PBMC transcription as a whole (with the exception of the study focused on B cells), we chose to compare highly enriched sorted lymphocyte subsets to gain further insight into JDM pathogenesis by delineating the role of each of these subsets at the transcript level.

Interestingly of the three lymphocyte subsets isolated from new-onset JDM patient PBMCs, NK cells had the highest number of differentially expressed genes compared to AMC (**Figure 1**) with little overlap with T and B cells (**Figure 2**). This suggests the immune dysregulation in new-onset JDM patients may uniquely impacts NK cells on a greater scale than the other lymphocyte subsets. We were able to confirm our prior results demonstrating the highly activated and proliferative phenotype of NK cells in new-onset JDM (20) by the upregulation of cell cycle and immune response related genes (**Table 2** and **Figure 3**). It is currently unknown if this is due to a prolonged and/or inefficient immune response during a viral infection, but we and others have confirmed an upregulation of type I IFN related genes as well (24).

Additionally, we report a reduction in ER signaling (**Table 2**) and a previously undescribed decrease in ribosomal RNA levels (**Figure 3C**) in NK cells from new-onset JDM patients compared to healthy pediatric controls. While ER stress has been suggested to play a role in inclusion body myositis (58), the role of ribosomes and ER stress in JDM has not been previously investigated. Decreased ribosomal RNA levels were observed in NK cells but not in T or B cells from new-onset JDM patients (**Figure 4**). It has been previously shown that cell cycle arrest leads to an increase in ribosome biogenesis (59), but our data suggests prolonged cell cycle activation in NK cells from JDM patients may lead to decreased ribosome biogenesis in NK cells.

NK cells differed from B and T cells in JDM patients in that they had both an upregulation of cell cycle genes and a downregulation of genes associated with ribosome biogenesis. While these two phenotypes seem to be distinct, they may be interrelated. Recently using scRNAseq, Smith et al. identified a small population of peripheral blood NK cells that were terminally differentiated and activated but had a loss of ribosomal expression in three healthy adult donors (25). Furthermore, when cells are highly activated, nutrients become scarce (60). Under these circumstances, ribosomes are a major source of amino acids and nucleotides (61), due to the fact that ribosomes account for almost half of all cellular proteins in proliferating NK cells and that ribosome biogenesis and protein translation are some of the most energy consuming cellular processes (62). While this unique low ribosomal NK subset accounted for less than 2% of the total NK cell population in healthy adult controls in the original report (25) and ~3% in healthy adult controls in our hands (**Figure 5A**), our work demonstrates that it makes up a much larger percentage (16% – 48%) of the NK cell population in new onset/treatment-naïve JDM patients (**Figure 6A**). We also show that low ribosome expressing NK cells have decreased CD107a degranulation in response to target cells compared to conventional NK cells from healthy adult donors (**Figure 5E**), suggesting this population has decreased NK cell functionality.

Previously, it has been shown that a defect in ribosome biogenesis resulted in NK cell dysfunction and increased susceptibility to cancer in a murine model (41). Recently, the inhibition of ribosomes in T cells (with the ribosome-targeting antibiotic Linezolid) has been shown to reduce T cell-mediated autoimmunity by impairing cytokine production and functionality within these cells (63). Our experiments demonstrate that new-onset and treatment naïve JDM patients have a substantially larger population of low ribosome expressing NK cells compared to healthy controls (**Figure 6A**), which correlates with decreased NK cell degranulation in the JDM patients (**Figure 6E**). The increased population of low ribosome expressing NK cells in JDM patients may also potentially contribute to other NK cell dysfunction in JDM patients, including suppressed PLCγ2 phosphorylation and subsequent calcium flux (20). Additionally, decreased ribosomal phosphorylation recovered in JDM patients with clinically inactive disease, along with NK cell percentages and NK cell degranulation (**Figure 7**). Together, these results demonstrate that altered ribosome biogenesis may impact NK cell functional responses, potentially contributing to the onset of in disorders like JDM, potentially through the loss of NK cell mediated regulation of dysregulated immune cells (e.g., autoimmune T cells).

The decreased RPS6 phosphorylation in JDM NK cells (**Figure 6D**) suggests dysregulation in the mTORc1 pathway, which regulates several steps in ribosome biogenesis, including ribosomal RNA transcription, and synthesis of ribosomal proteins, and other components required for ribosome assembly (43). The mTOR pathway is essential for NK cell functional responses. Human NK cells stimulated with CD16 crosslinking upregulate phosphorylated RPS6, a readout of mTORc1 signaling, with increasing intensity of stimulation (64). As shown in a murine model, RPS6 phosphorylation was positively correlated with NK cell effector functions, including target NK cell killing, degranulation, and IFNγ production, while mTOR inhibition reduced these functions (65). Additionally, mTOR is required for signaling of the NK cell activating cytokine IL-15 in humans (66). These examples demonstrate that various NK cell functions and activating pathways are dependent on mTOR signaling. We have demonstrated that JDM NK cells have decreased mTORc1 signaling as assessed by RPS6 phosphorylation (**Figure 6D**) and hypothesize that this deficit may contribute to decreased NK cell degranulation (**Figure 6E**) and calcium flux (20). The cause of decreased mTOR signaling (and decreased ribosomal expression overall) in JDM NK cells is currently unknown, but it is likely contributing to the observed dysregulation of NK cell function in JDM and has potential implications for JDM pathogenesis.

Decreased ribosome biogenesis could also be secondary to increased ER stress within the JDM NK cells. ER stress occurs when protein folding becomes dysregulated, secondary to environmental stressors, such as calcium dysregulation, viral infection, or nutrient deprivation (67). To counteract the increased misfolded proteins, the unfolded protein response (UPR) is activated (68). The UPR is initiated to attempt to return the cell to homeostasis; however, if the UPR response is overwhelmed or prolonged, autophagy is initiated. As shown in **Table 2**, JDM patient NK cells have a significant downregulation in genes associated with protein targeting to the ER membrane. In adult dermatomyositis, polymyositis, and inclusion body myositis, muscle biopsies have shown elevated levels of ER stress markers (68). In our RNAseq analysis, we found EIF2AK2, or protein kinase R (PKR), a negative regulator of translation that is activated during oxidative and ER stress (69), to be significantly upregulated in JDM NK cells (logFC = 2.022 and P Value = 0.000873). Additionally, we found GO value for “SRP-dependent co-translational protein targeting to membrane” is selectively downregulated in new-onset JDM NK cells (**Table 2**). SRP is downstream of PLCγ2 and required for IP_3_ signaling to the ER for calcium release (70). Its downregulation may contribute to the dampened NK cell calcium flux observed in treatment-naïve JDM patients and PLCγ2 dysregulation (20). This suggests ER stress from highly activated NK cells in JDM patients could lead to decreased ribosome biogenesis and a possible explanation for decreased calcium flux in JDM patients.

We describe here for the first time the dysregulation of ribosomes in JDM NK cells and propose that this may lead to further investigation of previously unstudied pathways in JDM. mTOR activation, a measurement of ribosome biogenesis, has been reported in a variety of chronic inflammatory diseases, including SLE and RA (71). In JDM NK cells, prolonged, activation and proliferation, and subsequent mTOR activation may result in decreased functionality of these cells, perhaps partially due to energy stores within cells being depleted. Whether decreased mTOR signaling is the main culprit of JDM NK cell dysregulation or is a consequence of an unknown upstream component that also affects this pathway remains to be determined. Future directions will focus on delineating the mechanism by which mTOR signaling is inhibited in JDM NK cells and determining if this can be modulated by specific inhibitors or activators for potential therapeutics.

The JDM patients used in these studies were all new onset with all but one (patient 3) being treatment naïve or in clinical remission on medication. The exclusion of patient 3 in an RNAseq sub-analysis did alter the number of differentially expressed genes identified in the three immune subsets; however, this did not alter the overall discovery of low ribosome expression being significantly downregulated in JDM NK cells as measured by the GO and WGCNA values determined in both analyses. While steroid treatment can alter the mTOR pathway (72), the dose and duration of steroid therapy necessary to suppress the mTOR pathway or NK cell function is not well characterized. Regardless the same conclusions regarding ribosome expression being down regulated can be made with or without the inclusion of this patient in the RNAseq analysis. Additionally, this patient was excluded from the remainder of the experiments identifying and defining function of the low ribosome NK population in treatment-naïve patients and did not interfere with those results.

This work provides novel, potential mechanistic insight into JDM pathogenesis. By performing RNAseq on sorted live lymphocytes, we were able to extend our previous results regarding NK cell hyperactivation and decreased NK cell function in JDM patients and identify an expanded, novel NK cell population with decreased ribosomal biogenesis which correlated with decreased NK cell effector functions. Future work will focus on further clarifyion of these alterations in NK cells from JDM patients to help delineate the mechanism causing the decreased mTOR signaling and suppressed ribosomal biogenesis. A better understanding of NK cell dysfunction in the pathogenesis of JDM in new-onset patients may facilitate novel therapeutic interventions to mitigate the impact of JDM on children.

## Data availability statement

The RNA-sequencing data has been made publicly available in the National Center for Biotechnology Information (NCBI) Gene Expression Omnibus (GEO) database under accession GSE211311.

## Ethics statement

The studies involving human participants were reviewed and approved by Human Research Protection Office At WAshintogn University in St Louis. Written informed consent to participate in this study was provided by the participants’ legal guardian/next of kin.

## Author contributions

KH and AF wrote and edited the manuscript. KH, AT and AF designed the research studies. KH, AT, and JP conducted experiments. KH and AF analyzed and interpreted data. NS and JP processed the samples. HZ provided ribosome expertise and guidance on the design of the confirmation studies. All authors contributed to the article and approved the submitted version.

## Funding

The MGI is partially supported by NCI Cancer Center Support Grant #P30 CA91842 to the Siteman Cancer Center and by ICTS/CTSA Grant# UL1TR002345 from the National Center for Research Resources (NCRR). We would also like to thank Madhurima Kaushal and Shamin Mollah from the Institute for Informatics (I^2^) at Washington University School of Medicine for guidance on the RNAseq analysis. We also thank Dr. Megan Cooper and the Center for Pediatric Immunology at St. Louis Children’s Hospital and Washington University for providing several of the age-matched controls. This work was supported by a Cure JM grant (ARF) and the WU-Rheumatic Diseases Research Resource-Based Center *(*P30AR073752).

## Acknowledgments

We thank Eric Tycksen at the Genome Technology Access Center at the McDonnell Genome Institute in the Department of Genetics at Washington University School of Medicine for help with genomic analysis.

## Conflict of interest

The authors declare that the research was conducted in the absence of any commercial or financial relationships that could be construed as a potential conflict of interest.

## Publisher’s note

All claims expressed in this article are solely those of the authors and do not necessarily represent those of their affiliated organizations, or those of the publisher, the editors and the reviewers. Any product that may be evaluated in this article, or claim that may be made by its manufacturer, is not guaranteed or endorsed by the publisher.
