# Supplementary material for: Expansion of a novel population of NK cells with low ribosome expression in juvenile dermatomyositis
Source: Front Immunol. 2022 Oct 31;13:1007022. doi: 10.3389/fimmu.2022.1007022 (PMC9660249; doi:10.3389/fimmu.2022.1007022)
Supplement: Supplementary file 1 [file DataSheet_1.docx]

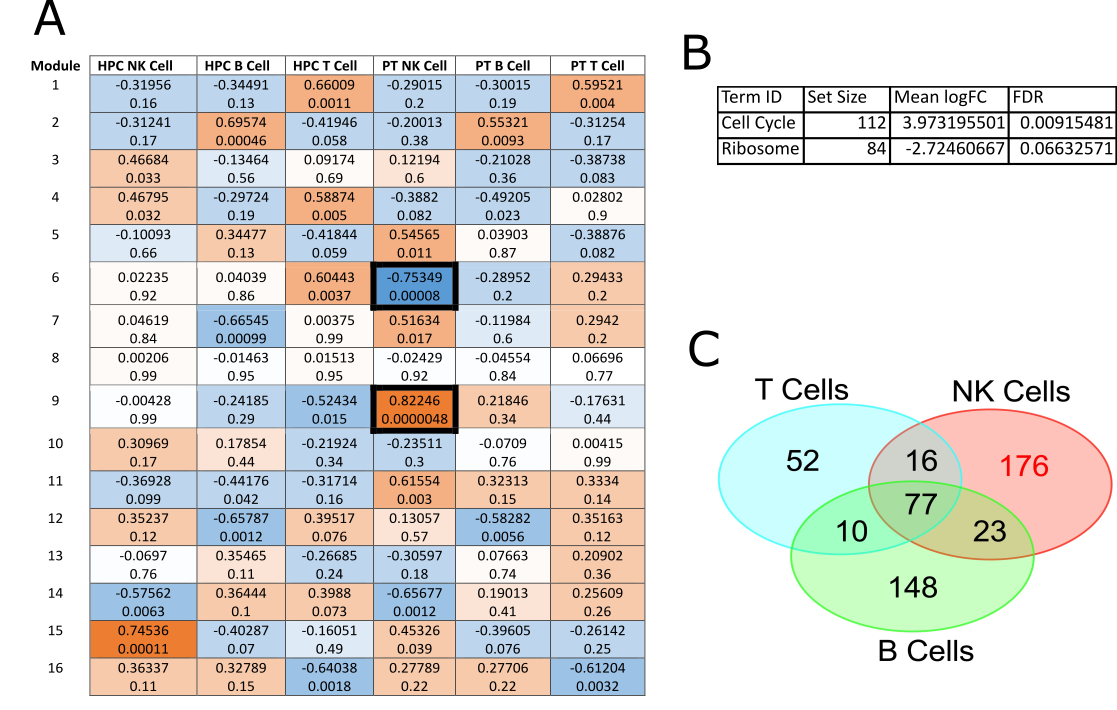


**Supplemental Figure 1: RNAseq analysis without JDM patient 3.** The RNAseq analysis was redone excluding JDM patient 3, who received initiation of treatment prior to his initial blood draw. A. The module trait and significance chart from the WGCNA. The top value for each module is the Spearman Correlation Coefficient and the bottom value is the P value. Blue indicates a negative correlation, while orange indicates a positive correlation with color intensity representing the correlation coefficient value. Module 9 here correlates with module 7 in Table 3 of the initial analysis, while module 6 correlates with module 1 in Table 3 of the initial analysis. B. The results from the GAGE KEGG Canonical Pathway gene sets for Cell Cycle and Ribosome (with patient 3 omitted). C. The overlap of differentially expressed genes among the cell types in JDM patients compared to age-matched controls (with patient 3 omitted. Differentially expressed genes were defined by a logFC >2 or <-2 and an adjusted P value of < 0.05.


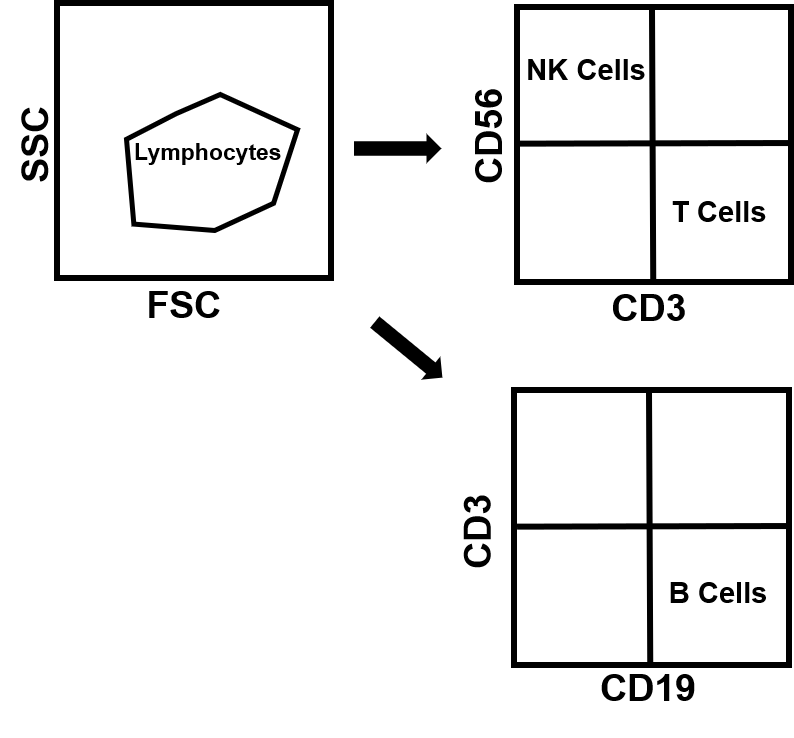


**Supplemental Figure 2: Gating strategy for sorting lymphocytes.** Flow cytometry gating strategy when sorting PBMCs for RNAseq.

**
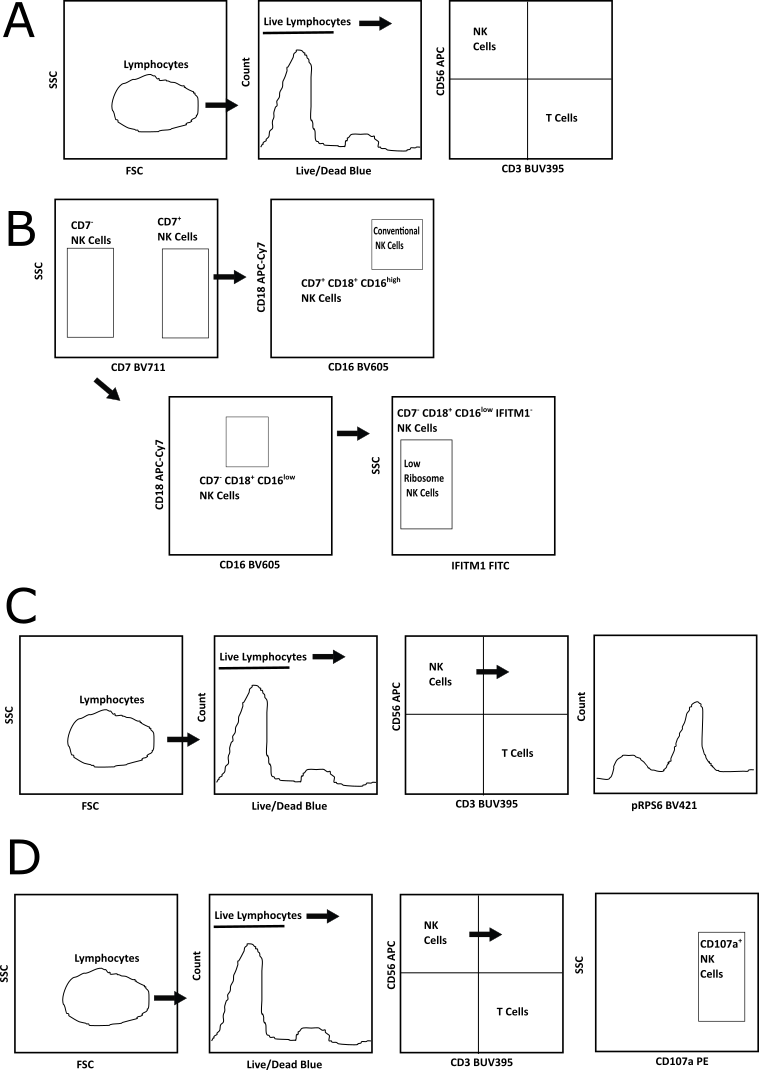
**

**Supplemental Figure 3: Gating strategies for flow cytometry experiments.** A. Gating strategy for determining live NK cells. B. Gating strategy for low ribosome expressing NK cells and the conventional population of NK cells used in Figure 5. C. Gating strategy for determining pRPS6 MFI. D. Gating strategy for determining NK cell degranulation.

**
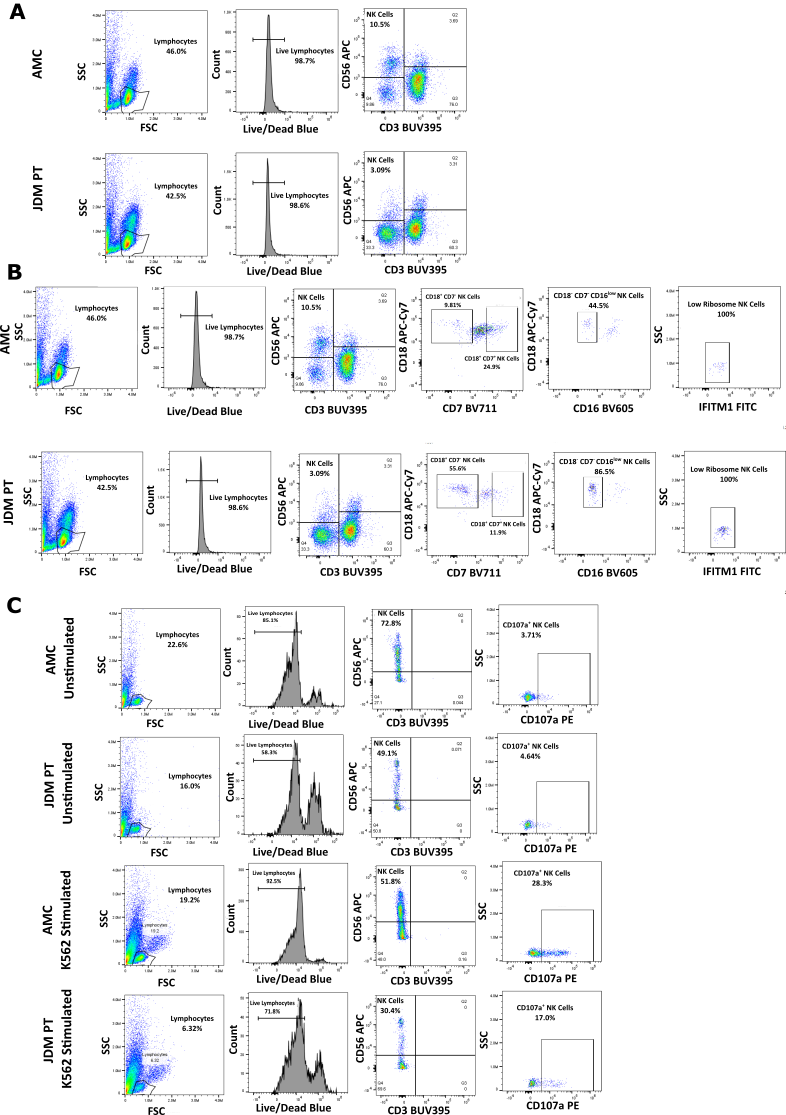
**

**Supplemental Figure 4: Representative flow plots for flow cytometry experiments.** A. Representative flow plots for determining NK cell percentages in JDM PT and AMC. B. Representative flow plots for determining the percentage of low ribosome NK cells in JDM PT and AMC. C. Flow plots for determining NK cell degranulation in enriched NK cells in a representative JDM PT and its corresponding AMC.


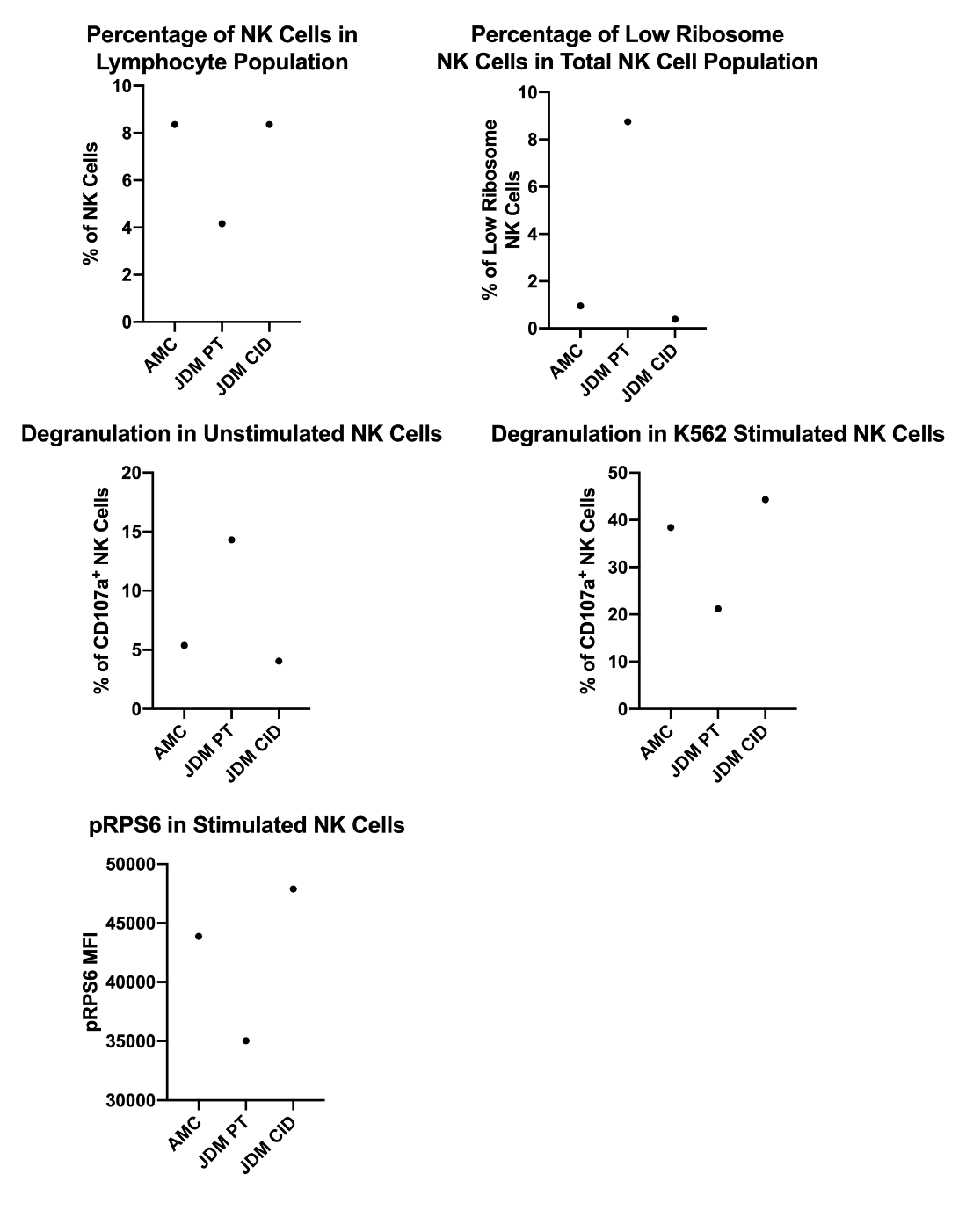


**Supplemental Figure 5: JDM Patient 6 pretreatment and with clinically inactive disease.** Top line: the percentage of NK cells in the total PBMC population and Low ribosome NK cells in the NK cell population in age-matched controls (AMC), JDM patient PT 6 (treatment naive), and JDM PT 6 when in clinically inactive disease (CID). Center line: NK Cell degranulation in enriched NK cells unstimulated or stimulated with K562 cells. Bottom line: pRPS6 MFI pRPS6 as measured by flow cytometry in stimulated, enriched NK cells.


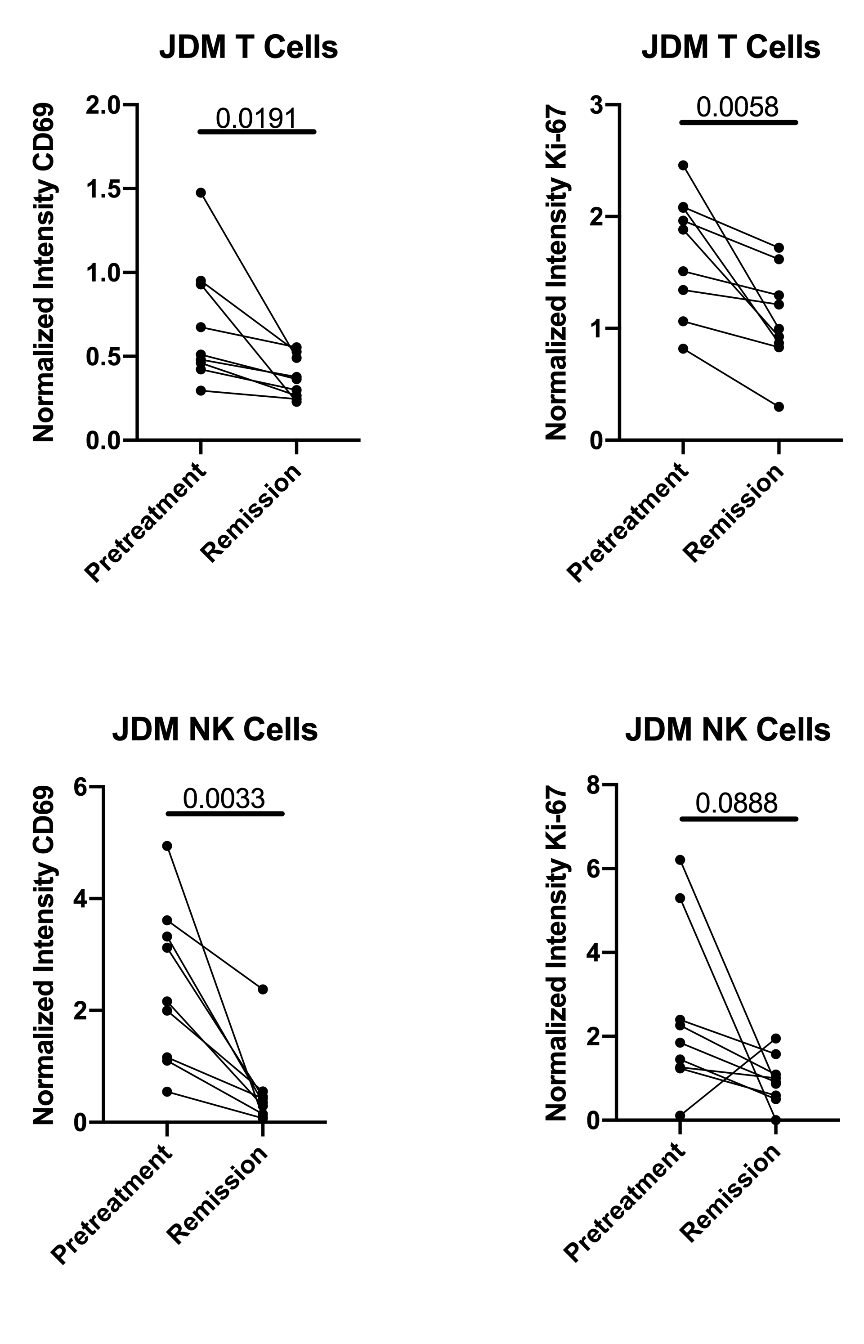


**Supplemental Figure 6: Activation and proliferation of JDM T and NK cells pretreatment and in remission.** A. T cells normalized intensity of CD69 (activation) and Ki-67 (proliferation) in JDM patients during pretreatment and in remission as measured by CyTOF. B. NK cells normalized intensity of CD69 and Ki-67 in JDM patients during pretreatment and in remission. This is previously unpublished data from our JDM CyTOF study (20).


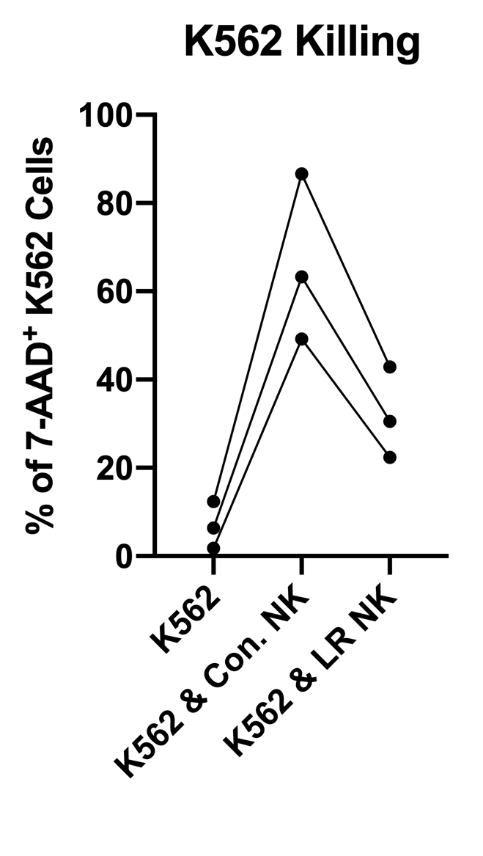


**Supplemental Figure 7: NK Cell Killing of K562 Cells in Control NK Cells and Low Ribosome Expressing NK Cells from healthy adult donors.**

Sorted control NK cells (CD7+, CD16high, and CD18+) and Low ribosome expressing NK cells (CD7-, CD16low,

CD18+, and IFITM1-) were co-cultured at a 1:1 ratio with violet labeled K562 cells. The percentage of dead K562

cells (7AAD+, Violet+) was determined by flow cytometry after 4 hours. These samples represent the the

average of NK cells 3 different LRS chambers (healthy adult donors).

| **PT** | **JDM Presentation and Duration of untreated disease at diagnosis (months)** | **Age at diagnosis or *Age of particpants Duration of untreated disease at diagnosis* (months)** | ***Age of particpants with quiescent disease* (yrs)** |
| --- | --- | --- | --- |
| 1 | Progressive weakness w/ difficulty going up stairs and erythematous rash over hands with Gottron’s papules 3 | 3 |  |
| 2 | Progressive weakness with classic Gottron’s palpules and elevated muscle enzymes. <3 | <3 |  |
| 2b | Currently doing well on low dose MMF, HCQ, and IVIG (q7 wk) |  | *14.2* |
| 3 | Progressive weakness, weight loss, Gottron’s papules, and scaly erythematous rash 4 | 4 |  |
| 4 | Progressive weakness with Gottron’s papules and elevated muscle enzymes (CK 7788) <1 | <1 |  |
| 5 | Photosensitive erythematous rash with progressive muscle weakness & modestly elevated muscle enzymes 4 | 4 |  |
| 6 | Muscle weakness, heliotrope rash, Gottron’s papules, and elevated muscle enzymes 1 | 1 |  |
| 6b | Currently doing well on a modest dose of MTX and just discontinued her q8 wk IVIG |  | *9.8* |
| 7 | Photosensitive rash >1 yr. Gottron’s papules, heliotrope rash, borderline CK with minimal myositis identified <1 | <1 |  |
| 8 | Progressive weakness and rash over his knees, elbows, and eyelids. Elevated muscle enzymes. Weakness worsened to the point he refused to walk or sit independently. 3 | <1 |  |
| 9 | Photosensitive rash, proximal muscle weakness, with elevated muscle enzymes. Currently doing well on IVIG, MMF, and HCQ 2 | 2 | *10.3* |
| 10 | Progressive weakness with rash over knuckles/hands, feet and face with elevated muscle enzymes. Due to difficulty weaning steroids, she received a 2-dose course of rituximab 7 mo ago. Currently doing well on IVIG (q7 wk), Cellcept, and HCQ 1-2 | 1.5 | *8.7* |

**Supplemental Table 1: Patient presentation and medication regimen.** JDM presentation and duration of untreated disease in all patients with medication regimen stated for the four clinically inactive disease patients. Patients were diagnosed with JDM based on modified Bohan and Peter’s criteria 1) symmetric, proximal muscle weakness, 2) characteristic rash (e.g, heliotrope rash, Gottron’s papules, shawl sign, or periungual telangiectasias), 3) elevated muscle enzymes, 4) presence of myositis-specific autoantibodies, and 5) other findings (calcinosis, dysphagia, or skin ulcerations) in a manner very similar to the Myositis Association’s JDM diagnostic criteria. More detailed specialized scales such as CMAS (childhood myositis assessment scale) or MMT-8 (manual muscle testing of 8 muscle groups) were not routinely used.

Drug definitions: MMF- mycophenolate mofetil also know as Cellcept, HCQ -hydroxychloroquine, IVIG -intervenous immunoglobulin, MTX - methotrexate

**Upregulated Overlapping DE Genes**

| **NK, B, & T** | **NK & B** | **NK & T** | **T & B** |
| --- | --- | --- | --- |
| CMPK2 | IFI44 | OAS1 | DDX60 |
| EIF2AK2 | MX2 | EXO1 | XAF1 |
| EPSTI1 | HERC5 |  |  |
| IFI44L | HERC6 |  |  |
| IFIT1 | IFI6 |  |  |
| IFIT3 | LGALS3PB |  |  |
| ISG15 | STAT1 |  |  |
| MX1 | U62317.4 |  |  |
| OAS2 |  |  |  |
| OASL |  |  |  |
| PARP9 |  |  |  |
| PLSCR1 |  |  |  |
| RSAD2 |  |  |  |
| SPATS2L |  |  |  |
| USP18 |  |  |  |

**Downregulated Overlapping DE Genes**

| **NK, B, & T** | **NK & B** | **NK & T** | **T & B** |
| --- | --- | --- | --- |
|  | NT5C3B | \| AC006369.1 \| \| --- \| |  |
|  |  | C17orf100 |  |
|  |  | CD160 |  |

**Supplemental Table 2: Overlapping upregulated and downregulated DE genes in NK, T, and B cells in JDM patients.** List of DE genes that significantly upregulated or downregulated in JDM patients in multiple lymphocyte groups compared to healthy pediatric controls.

| **Accession** | **Description** | **Number of Genes** | **logFC** | **P value** |
| --- | --- | --- | --- | --- |
| GO:0051276 | Chromosome organization | 991 | 6.959626 | 2.34E-12 |
| GO:0006259 | DNA metabolic process | 833 | 6.363444 | 1.29E-10 |
| GO:0006396 | RNA processing | 805 | 6.026044 | 1.15E-09 |
| GO:0051607 | Defense response to virus | 185 | 5.798839 | 8.23E-09 |
| GO:0006281 | DNA repair | 479 | 5.782777 | 5.16E-09 |
| GO:0000278 | Mitotic cell cycle | 818 | 5.739334 | 5.67E-09 |
| GO:0009615 | Response to virus | 243 | 5.576955 | 2.18E-08 |
| GO:0034660 | NcRNA metabolic process | 502 | 5.568643 | 1.80E-08 |
| GO:1903047 | Mitotic cell cycle process | 712 | 5.539906 | 1.80E-08 |
| GO:0006996 | Organelle organization | 2906 | 5.497939 | 2.01E-08 |
| GO:0007049 | Cell cycle | 1425 | 5.467981 | 2.48E-08 |
| GO:0034340 | Response to type I interferon | 70 | 5.412568 | 2.01E-07 |
| GO:0060337 | Type I interferon signaling pathway | 66 | 5.364064 | 2.78E-07 |
| GO:0071357 | Cellular response to type I interferon | 66 | 5.364064 | 2.78E-07 |
| GO:0007059 | Chromosome segregation | 265 | 5.299234 | 8.60E-08 |
| GO:0000819 | Sister chromatid segregation | 166 | 5.265827 | 1.26E-07 |
| GO:0071103 | DNA conformation change | 258 | 5.05692 | 3.07E-07 |
| GO:0022402 | Cell cycle process | 1065 | 5.006817 | 3.00E-07 |
| GO:0044772 | Mitotic cell cycle phase transition | 478 | 4.943549 | 4.55E-07 |
| GO:0006974 | Cellular response to DNA damage stimulus | 727 | 4.841597 | 7.17E-07 |
| GO:0000070 | Mitotic sister chromatid segregation | 140 | 4.648235 | 2.60E-06 |
| GO:0006325 | Chromatin organization | 629 | 4.64092 | 1.93E-06 |
| GO:0098813 | Nuclear chromosome segregation | 212 | 4.631563 | 2.42E-06 |
| GO:0044770 | Cell cycle phase transition | 507 | 4.612418 | 2.25E-06 |
| GO:0022613 | Ribonucleoprotein complex biogenesis | 422 | 4.417765 | 5.98E-06 |
| GO:0002274 | Myeloid leukocyte activation | 512 | -4.70371 | 1.46E-06 |
| GO:0001775 | Cell activation | 1017 | -4.62194 | 2.02E-06 |
| GO:0042119 | Neutrophil activation | 408 | -4.55697 | 3.01E-06 |
| GO:0002263 | Cell activation involved in immune response | 563 | -4.5118 | 3.56E-06 |
| GO:0002366 | Leukocyte activation involved in immune response | 560 | -4.45959 | 4.53E-06 |
| GO:0036230 | Granulocyte activation | 413 | -4.36849 | 7.09E-06 |
| GO:0043299 | Leukocyte degranulation | 433 | -4.32652 | 8.50E-06 |
| GO:0002443 | Leukocyte mediated immunity | 605 | -4.31983 | 8.48E-06 |
| GO:0002275 | Myeloid cell activation involved in immune response | 438 | -4.27871 | 1.05E-05 |
| GO:0045055 | Regulated exocytosis | 572 | -4.15155 | 1.78E-05 |
| GO:0002444 | Myeloid leukocyte mediated immunity | 448 | -4.15082 | 1.82E-05 |
| GO:0045321 | Leukocyte activation | 927 | -4.08208 | 2.33E-05 |
| GO:0007186 | G protein-coupled receptor signaling pathway | 365 | -4.03441 | 3.05E-05 |
| GO:0046903 | Secretion | 1039 | -3.82249 | 6.81E-05 |

**Supplemental Table 3: Top upregulated and downregulated B cell GO values in JDM patients.** List of the top 25 significantly upregulated and the 14 significantly downregulated Biological Process GO categories in new-onset JDM B cells compared to healthy pediatric controls.

| **Accession** | **Description** | **Number of Genes** | **logFC** | **P value** |
| --- | --- | --- | --- | --- |
| GO:0051276 | Chromosome organization | 991 | 4.63221 | 1.94E-06 |
| GO:0034340 | Response to type I interferon | 70 | 4.359836 | 1.51E-05 |
| GO:0060337 | Type I interferon signaling pathway | 66 | 4.350689 | 1.65E-05 |
| GO:0071357 | Cellular response to type I interferon | 66 | 4.350689 | 1.65E-05 |

**Supplemental Table 4: Top upregulated T cell GO values in JDM patients.** List of the top significantly upregulated Biological Process GO categories in new-onset JDM T cells compared to healthy pediatric controls. There were no significantly downregulated categories.

| **External Gene Name** | **Correlation Module Membership** | **P value Module Membership** |
| --- | --- | --- |
| TIMM17A | 0.940230418 | 9.08E-12 |
| TMED5 | 0.939514124 | 1.03E-11 |
| TXNDC17 | 0.934571126 | 2.39E-11 |
| MCM10 | 0.925873067 | 9.06E-11 |
| AURKB | 0.923733212 | 1.23E-10 |
| CDK1 | 0.922743967 | 1.41E-10 |
| SRP54 | 0.92007619 | 2.02E-10 |
| CASP3 | 0.917874517 | 2.69E-10 |
| RRM2 | 0.911568986 | 5.89E-10 |
| NOP10 | 0.906732481 | 1.03E-09 |
| NUSAP1 | 0.905315709 | 1.21E-09 |
| TYMS | 0.902942579 | 1.57E-09 |
| PPP2CA | 0.900089848 | 2.14E-09 |
| BIRC5 | 0.899201026 | 2.34E-09 |
| ESCO2 | 0.89915755 | 2.36E-09 |
| DYNLL1 | 0.899016582 | 2.39E-09 |
| H2BC4 | 0.897625792 | 2.76E-09 |
| POMP | 0.897130935 | 2.90E-09 |
| NIPSNAP3A | 0.894002755 | 3.98E-09 |
| UBE2A | 0.892453473 | 4.63E-09 |

**Supplemental Table 5: Top 20 Correlation Module Membership genes in Module 7.** Genes with the highest correlation module membership in module 7 of the WGCNA analysis. These genes are the best representation of the set of the remaining genes (369 genes total). Yellow indicates association with cellular activation, while blue indicates association with cellular proliferation.

| **External Gene Name** | **Correlation Module Membership** | **P value Module Membership** |
| --- | --- | --- |
| RPL12 | 0.990786128 | 1.34E-20 |
| RPL23 | 0.979921627 | 6.71E-17 |
| RPLP2 | 0.978114093 | 1.72E-16 |
| RPL37 | 0.974062451 | 1.09E-15 |
| RPS15 | 0.972711883 | 1.90E-15 |
| RPL39 | 0.971967389 | 2.54E-15 |
| AP001324.1 | 0.971895336 | 2.61E-15 |
| RPL11 | 0.971181436 | 3.43E-15 |
| AC112491.1 | 0.970921184 | 3.79E-15 |
| RPS21 | 0.970538927 | 4.36E-15 |
| RPS13 | 0.970379372 | 4.63E-15 |
| FO393411.1 | 0.969764468 | 5.78E-15 |
| RPS20 | 0.969741522 | 5.83E-15 |
| RPL18AP3 | 0.969296135 | 6.83E-15 |
| RPL36 | 0.968602851 | 8.71E-15 |
| RPL23A | 0.968407454 | 9.32E-15 |
| RPS23 | 0.968034762 | 1.06E-14 |
| RPS15P4 | 0.966915077 | 1.54E-14 |
| IMPDH2 | 0.965932943 | 2.11E-14 |
| RHOH | 0.965679215 | 2.29E-14 |

**Supplemental Table 6: Top 20 Correlation Module Membership genes in Module 1.** Genes with the highest correlation module membership in module 1 of the WGCNA analysis. These genes are the best representation of the set of the remaining genes (1578 genes total). Yellow indicates association with ribosomal proteins.

**Cell Cycle Genes**

| ABL1 | CCNA2* | CDC25A* | CDKN2C* | GADD45A* | MDM2* | RBL1 | TFDP2 |
| --- | --- | --- | --- | --- | --- | --- | --- |
| ANAPC1 | CCNB1* | CDC25B | CDKN2D | GADD45B* | MYC | RBL2* | TGFB1 |
| ANAPC10 | CCNB2* | CDC25C* | CHEK1 | GSK3B | ORC1* | RBX1* | TGFB2 |
| ANAPC11 | CCNB3 | CDC26 | CHEK2* | HDAC1 | ORC2 | SKP1V* | TGFB3 |
| ANAPC13 | CCND2 | CDC27 | CREBBP | HDAC2 | ORC3 | SKP2 | TP53V* |
| ANAPC2 | CCND3 | CDC45* | CUL1 | MAD1L1 | ORC4 | SMAD2* | TTK |
| ANAPC4 | CCNE1 | CDC6 | DBF4 | MAD2L1* | ORC5 | SMAD3 | WEE1 |
| ANAPC5* | CCNE2 | CDC7* | E2F1 | MAD2L2 | ORC6* | SMAD4 | YWHAB |
| ANAPC7 | CCNH | CDK1V* | E2F2* | MCM2* | PCNA* | SMC1A | YWHAE* |
| ATM* | CDC14A | CDK2* | E2F3* | MCM3 | PLK1 | SMC1B | YWHAG |
| ATR | CDC14B* | CDK4 | E2F4 | MCM4* | PRKDC | SMC3 | YWHAH |
| BUB1V | CDC16 | CDK6 | E2F5 | MCM5* | PTTG1* | STAG1 | YWHAQ |
| BUB1B* | CDC20 | CDKN1A | EP300 | MCM6* | RAD21 | STAG2 | YWHAZ |
| BUB3 | CDC23 | CDKN1B* | FZR1 | MCM7* | RB1 | TFDP1* | ZBTB17 |

**Ribosome Genes**

| FAU | RPL18* | RPL27* | RPL36 | RPL7A | RPS15A* | RPS25* | RPS5* |
| --- | --- | --- | --- | --- | --- | --- | --- |
| MRPL13* | RPL18AV | RPL27A* | RPL36A | RPL8 | RPS16* | RPS26 | RPS6* |
| RPL10* | RPL19* | RPL28 | RPL36AL | RPL9 | RPS17 | RPS27* | RPS7* |
| RPL10A* | RPL21 | RPL29 | RPL37* | RPLP0* | RPS17 | RPS27A* | RPS8* |
| RPL11* | RPL22* | RPL3* | RPL37A* | RPLP1 | RPS18 | RPS27L* | RPSA |
| RPL12* | RPL22L1 | RPL30* | RPL38 | RPLP2* | RPS19 | RPS28 | RSL24D1 |
| RPL13* | RPL23 | RPL31 | RPL39 | RPS10 | RPS2 | RPS29* | UBA52* |
| RPL13A* | RPL23A | RPL32V | RPL4* | RPS11V* | RPS20* | RPS3* |  |
| RPL14* | RPL24* | RPL34* | RPL41* | RPS12 | RPS21 | RPS3A* |  |
| RPL15* | RPL26* | RPL35* | RPL5* | RPS13 | RPS23* | RPS4X* |  |
| RPL17 | RPL26L1* | RPL35A* | RPL7 | RPS15 | RPS24* | RPS4Y1 |  |

**Supplemental Table 7: List of genes in the KEGG Cell Cycle and Ribosome Pathways.** The list of Cell Cycle and Ribosome genes shown in the volcano plots in Figure 4. These are the same genes depicted in the heat maps in Figure 3. The genes that remained significant after multiple hypothesis corrections are denoted by a red asterisks (*).
